# Chronic Plasma Cortisol Elevation Does Not Promote Riskier Behavior in a Teleost Fish: A Test of the Behavioral Resiliency Hypothesis

**DOI:** 10.1093/iob/obz009

**Published:** 2019-04-26

**Authors:** Michael J Lawrence, Jean-Guy J Godin, Aaron J Zolderdo, Steven J Cooke

**Affiliations:** 1 Fish Ecology and Conservation Physiology Laboratory, Department of Biology, Carleton University, Ottawa, Ontario, Canada K1S 5B6; 2 Department of Biology, Carleton University, Ottawa, Ontario, Canada K1S 5B6; 3 Queen’s University Biological Station, Queen’s University, Elgin, Ontario, Canada K0G 1E0

## Abstract

Stressed fish have been shown to have higher predator-induced mortality than unstressed conspecifics, suggesting a role for the hypothalamic–pituitary–interrenal axis in modifying risk-taking behaviors. Yet, there is also evidence of behavioral resiliency in the face of chronic stressors. Here, we tested the behavioral resiliency hypothesis, which posits that animals can maintain consistent behavioral phenotypes in the face of significant physiological challenges. We determined whether chronic plasma cortisol elevation promotes risk-taking behaviors in a model teleost fish, the pumpkinseed sunfish (*Lepomis gibbosus*). Experimental fish were implanted with cocoa butter either as a sham or with cortisol. At 48 h post-implantation, the behavior of individual focal fish was tested in an experimental arena comprising of a simulated physical refuge, an open zone containing a constrained conspecific shoal, and a compartment containing either a model of a northern pike (*Esox lucius*) paired with corresponding pike olfactory cues in lake water or no pike model (control) paired with sham lake water cues only. The fish were assayed individually for their refuge utilization, shoaling tendency, and general activity. Neither cortisol treatment nor predation-risk treatment influenced any of these behaviors. This suggests that sunfish, in the context of our experiment, were behaviorally resilient to the physiological effects of chronic plasma cortisol elevation and in the face of an apparent threat of predation. Our results thus provide support for the behavioral resiliency hypothesis in fish under both physiological and ecological stressors. We posit that behavioral resiliency is an evolutionary adaptation ensuring appropriate responses to environmental conditions.

## Synopsis

Elevação Crônica do Cortisol Plasmático não Promove Comportamento Mais Arriscado em um Peixe Teleósteo: Um teste da Hipótese de Resiliência Comportamental (Chronic Plasma Cortisol Elevation Does Not Promote Riskier Behavior in a Teleost Fish: A Test of the Behavioral Resiliency Hypothesis) Foi demonstrado que peixes estressados têm maior mortalidade causada por predadores do que co-específicos não estressados, sugerindo um papel para o eixo hipotálamo–hipófise–interrenal na modificação de comportamentos de risco. No entanto, há também evidências de resiliência comportamental frente a estressores crônicos. Aqui testamos a hipótese de resiliência comportamental, que postula que os animais podem manter fenótipos comportamentais consistentes frente a desafios fisiológicos significativos. Determinamos se a elevação crônica do cortisol plasmático promove comportamentos de risco em um peixe teleósteo modelo, o perca-sol (*Lepomis gibbosus*). Peixes experimentais foram implantados com manteiga de cacau, tanto como um placebo ou com cortisol. Após 48 h da implantação, o comportamento de peixes individuais foi testado em uma arena experimental composta por um refúgio simulado, uma zona aberta contendo um cardume co-específico e um compartimento contendo um modelo de lúcio-do-norte (*Esox lucius*) pareado com as pistas olfativas correspondentes de lúcios em água fluvial ou sem modelo (controle) pareado apenas com iscas falsas. Os peixes foram analisados individualmente quanto à utilização de refúgio, tendência de formar cardumes, e atividade geral. Nem o tratamento com cortisol e nem o com risco de predação influenciaram qualquer um desses comportamentos. Isto sugere que os perca-sol, no contexto do nosso experimento, eram comportamentalmente resilientes aos efeitos fisiológicos da elevação crônica do cortisol plasmático e diante de uma aparente ameaça de predação. Nossos resultados fornecem apoio para a hipótese de resiliência comportamental em peixes sob estressores fisiológicos e ecológicos. Nós postulamos que a resiliência comportamental é uma adaptação evolutiva que garante respostas apropriadas às condições ambientais. translated to Portuguese by G. Sobral (gabisobral@gmail.com)

La Elevación Crónica de Cortisol en el Plasma no Promueve un Comportamiento más Riesgoso en un Pez Teleósteo: Una Prueba de la Hipótesis de Resistencia de Comportamiento (Chronic Plasma Cortisol Elevation Does Not Promote Riskier Behavior in a Teleost Fish: A Test of the Behavioral Resiliency Hypothesis) Se ha demostrado que los peces estresados tienen una mayor mortalidad inducida por depredadores que los conespecíficos no estresados, lo que sugiere un papel para el eje hipotálamo–pituitario–interrenal en la modificación de los compartamientos de riesgo. Sin embargo, también hay evidencia de resistencia del comportamiento frente a los factores estresantes crónicos. Aquí, probamos la hipótesis de resistencia del comportamiento, que postula que los animales pueden mantener fenotipos de comportamiento consistentes ante desafíos fisiológicos significativos. Determinamos si la elevación crónica de cortisol en plasma promueve comportamientos de riesgo en un pez modelo teleósteo, el pez sol de semillas de calabaza (*Lepomis gibbosus*). Los peces experimentales se implantaron con manteca de cacao como una farsa o con cortisol. A las 48 h posteriores a la implantación, se evaluó el comportamiento de los peces focales individuales en un campo experimental que comprende un refugio físico simulado, una zona abierta que contiene un banco de peces conspecificos constreñidos, y un compartimento que contiene un modelo de lucio norteño (*Esox lucius*) emparejado con señales olfativas de lucio correspondientes en el agua del lago o sin modelo de lucio (control) emparejado solo con señales de agua del lago simulado. Los peces fueron analizados individualmente por su utilización de refugio, tendencia al cardumen y actividad general. Ninguno de estos comportamientos fuero influidos por el tratamiento con cortisol o el tratamiento de riesgo de depredación. Esto sugiere que los peces sol, en el contexto de nuestro experimento, eran resistentes al comportamiento frente a los efectos fisiológicos de la elevación crónica de cortisol en el plasma y ante una amenaza aparente de depredación. Por lo tanto, nuestros resultados brindan apoyo para la hipótesis de resistencia de comportamiento en peces bajo factores de estrés fisiológicos y ecológicos. Postulamos que la resiliencia conductual es una adaptación evolutiva que garantiza respuestas adecuadas a las condiciones ambientales. translated to Spanish by Y. E. Jimenez (yordano_jimenez@brown.edu)

Chronische Cortisol-Erhöhung im Plasma Fördert Kein Risikoreicheres Verhalten bei einem Echten Knochenfisch: Ein Test der Verhaltens-Resilienz-Hypothese (Chronic Plasma Cortisol Elevation Does Not Promote Riskier Behavior in a Teleost Fish: A Test of the Behavioral Resiliency Hypothesis) Es wurde gezeigt, dass gestresste Fische eine höhere durch Prädatoren induzierte Mortalität aufweisen als nicht gestresste Artgenossen, was auf eine Rolle der Hypothalamus–Hypophysen–Interrenalachse (Achse) bei der Änderung des Risikoverhaltens hindeutet. Es gibt jedoch auch Hinweise auf Verhaltensresilienz gegenüber chronischen Stressoren. Hier haben wir die Verhaltens-Resilienz-Hypothese getestet, die besagt, dass Tiere konsistente Verhaltensphänotypen angesichts erheblicher physiologischer Herausforderungen aufrechterhalten können. Wir haben ermittelt, ob eine chronische Cortisol-Erhöhung im Plasma das Risikobereitschaftsverhalten eines Modellfisches, des Gemeinen Sonnenbarsches (*Lepomis gibbosus*), fördert. Im Rahmen des Experiments wurde den Fischen Kakaobutter implantiert, zum Schein oder mit Cortisol. 48 Stunden nach der Implantation wurde das Verhalten einzelner zentraler Fische in einer experimentellen Arena getestet, bestehend aus einem simulierten physischen Zufluchtsort, einer offenen Zone mit einem eingeschränkten Schwarm der gleichen Art und einem Kompartiment mit entweder dem Modell eines Hechts (*Esox lucius*), gepaart mit entsprechenden olfaktorischen Signalen des Hechts im Wasser, oder ohne Hechtmodell (Kontrolle), gepaart nur mit Scheinsignalen. Die Fische wurden einzeln auf ihre Nutzung des Zufluchtsorts, Schwarmneigung und allgemeine Aktivität untersucht. Weder die Behandlung mit Cortisol noch die Prädationsrisiko-Behandlung beeinflussten diese Verhaltensweisen. Dies legt nahe, dass Sonnenbarsche im Rahmen unseres Experiments verhaltensresilient gegenüber den physiologischen Auswirkungen einer chronischen Cortisol-Erhöhung im Blut waren und angesichts einer scheinbaren Bedrohung durch Prädatoren. Unsere Ergebnisse unterstützen somit die Verhaltens-Resilienz-Hypothese in Bezug auf Fische unter sowohl physiologischen als auch ökologischen Stressfaktoren. Wir sind der Meinung, dass Verhaltensresilienz eine evolutionäre Anpassung ist, die angemessene Reaktionen auf Umweltbedingungen gewährleistet. translated to German by F. Klimm (frederike.klimm@neptun.uni-freiburg.de)

## Introduction

Predation risk, the probability of an organism succumbing to predation (i.e., P(death); Lima and Dill 1990), can have a profound impact on the life history and behavior of animals. Indeed, in teleost fishes, predators can reduce the foraging effort of prey fish (e.g., Werner et al. 1983; Milinski 1985; Mikheev et al. 2006), force prey to spend a greater amount of time in refuges (Werner et al. 1983; Gilliam and Fraser 1987; Krause et al. 1998), and reduce the general activity of prey (Bean and Winfield 1995; Pettersson et al. 2001; Laurel and Brown 2006). While these behavioral responses can minimize individual risk of predation, they are also associated with fitness costs in the form of lost opportunities (i.e., poor foraging, mating, etc.; Ydenberg and Dill 1986; Lima and Dill 1990). Thus, prey animals must balance predator-induced mortality risk with fitness-enhancing activities in such a way that the individual’s overall fitness is maximized (e.g., µ/g rule; Gilliam 1982; Werner and Gilliam 1984; Gilliam and Fraser 1987; Lima and Dill 1990).

The extent to which an individual accepts predation risk is highly contextual and is considered to be state dependent; a situation where the internal energetic/nutritional status dictates the acceptable level of predation risk and the associated behavioral phenotypes (reviewed in Godin 1997). State dependency has been shown to be an important regulator of risk-taking behaviors in teleost fishes wherein increasing energetic distress, often in the form of increasing hunger or metabolic loading, is associated with riskier behavioral phenotypes. For example, in Atlantic salmon (*Salmo salar*), hungrier fish resumed foraging activities sooner and had a greater foraging range than satiated specifics (Gotceitas and Godin 1991). Indeed, the influence of hunger on risk-taking behaviors appears to be ubiquitous across a number of model teleostean systems (e.g., Smith 1981; Dill and Fraser 1984; Godin and Smith 1988; Godin and Crossman 1994). Furthermore, higher resting metabolic rates (Krause et al. 1998, 2000; Dowling and Godin 2002) and parasite burden (Giles 1983, 1987; Milinski 1985; Godin and Sproul 1988) can promote riskier behavioral phenotypes, which generally includes reduced refuge usage, shorter post-attack behavioral latencies, and higher activity levels.

The general stress response also appears to be an important mediator of predation risk in teleosts. Under a broad range of contexts, species, and settings, teleost fish in a stressed state suffer higher rates of predation relative to unstressed conspecifics (reviewed in Mesa et al. 1994; Raby et al. 2014). Although the specific mechanism(s) underlying this pattern are currently unknown, the involvement of stressors in influencing individual susceptibility to predation risk implies a role for the hypothalamic–pituitary–interrenal (HPI) axis in mediating such interactions. Briefly, the HPI axis is one of the primary systems that re-establishes internal homeostasis following a physiological perturbation. HPI axis stimulation results in an increased biosynthesis of cortisol, the primary glucocorticoid hormone in teleosts (reviewed in Gorissen and Flik 2016; Schreck and Tort 2016). Cortisol generally increases energy substrate biosynthesis and availability, the temporary divestment of energetic resources away from fitness enhancing processes, and aids in re-establishing hydromineral balance (Mommsen et al. 1999; Schreck and Tort 2016). Together, this suite of metabolic responses ensures that the animal has sufficient resources to mitigate the effects of stressors, thereby adaptively maintaining internal homeostasis and steady-state conditions (Wendelaar Bonga 1997; Schreck and Tort 2016).

To date, few studies have attempted to study the direct role of cortisol in risk-taking and antipredator behaviors in teleost fish. However, several other vertebrate taxa have been investigated with respect to the effects of cortisol on antipredator and risk-taking behaviors. For example, in herpetofauna, application of exogenous glucocorticoids can enhance antipredator behaviors (Thaker et al. 2009, 2010; Trompeter and Langkilde 2011; Polich et al. 2018) and are regarded as important physiological mediators of threat perception (Thaker et al. 2010). However, this effect is not always consistent (Wack et al. 2013; Neuman-Lee et al. 2015). In mammals, treatment with glucocorticoids typically reduces risk-taking behaviors, which presumably would enhance predator avoidance in the wild (Murray et al. 2008; Rosen et al. 2008; Diniz et al. 2011). In comparison, the teleostean literature is rather scant with examples of cortisol-induced changes in risk-taking behaviors. The current body of work suggests that, despite significant physiological perturbations associated with cortisol’s actions, cortisol treatment appears to have little effect on behavioral measures of risk-taking and antipredator behaviors. For example, in schoolmaster snapper (*Lutjanus apodus*; Lawrence et al. 2017, 2018a), checkered pufferfish (*Sphoeroides testudineus*; Cull et al. 2015; Pleizier et al. 2015), pumpkinseed sunfish (*Lepomis gibbosus*; Lawrence et al. 2018b), and frillfin goby (*Bathygobius soporator*; specifically sheltering behavior; Barreto et al. 2014) cortisol treatment failed to affect antipredator and risk-taking behaviors. Furthermore, chronically-stressed zebrafish (*Danio rerio*) maintained a high degree of shoal cohesion for up to 7 days of stressor exposure, with cohesion breaking down thereafter (Piato et al. 2011), and they did not modify their feeding behavior (Pavlidis et al. 2015). Together, these results suggest that teleosts are able to demonstrate a relatively high degree of behavioral resiliency, that is, the maintenance of steady-state behavior under chronically-elevated plasma cortisol levels (e.g., Piato et al. 2011; Schmidt et al. 2017)—herein termed the “behavioral resilience hypothesis.” This hypothesis posits that the behavioral phenotypes of afflicted individuals should be comparable to the baseline state (i.e., not afflicted population), with the magnitude and direction of the responses across various environmental contexts (e.g., predation threats, social interactions, foraging activity, etc.) being conserved. For example, both stressed and unstressed conspecifics should exhibit comparable avoidance tactics when presented with a predatory threat if behavioral resiliency does indeed exist. Behavioral resiliency in the face of physiological perturbations likely serves as an adaptive mechanism ensuring that the animal behaviorally responds to ambient environmental stimuli in an appropriate manner to maximize fitness (Romero et al. 2009; Boonstra 2013a, 2013b; Sørensen et al. 2013). Failure to maintain “normal” behavior in physiologically stressed animals, in the context of predator–prey interactions, could result in sub-optimal fitness. A loss of behavioral resiliency/coping under stressor exposure may help explain why stressed fish experience higher rates of predation compared with unstressed conspecifics (Mesa et al. 1994; Raby et al. 2014). However, this notion of behavioral resiliency, in the context of cortisol-mediated chronic stress, has not been assessed to any great extent.

The purpose of our current study was to experimentally test the behavioral resiliency hypothesis. We suggest that the behavioral resiliency hypothesis posits that, in the face of physiological perturbations, individuals should be able to maintain a consistent behavioral phenotype that conceivably ensures high fitness. To do so, we evaluated whether wild-caught pumpkinseed sunfish (*L.gibbosus* Linnaeus 1758), used here as a model teleost fish, can exhibit behavioral resiliency with respect to risk-taking behaviors when their plasma cortisol levels are experimentally elevated. Given cortisol’s potential role in enhancing metabolic rate (De Boeck et al. 2001; O’Connor et al. 2010) and the role of energetic state in mediating risk-taking behaviors (e.g., Gotceitas and Godin 1991; Godin and Smith 1988; Skajaa et al. 2003; Killen et al. 2011), we expected that behavioral coping would not be possible in cortisol-treated fish who should therefore exhibit higher risk-taking behaviors than sham-treated conspecifics. Thus, we predicted that cortisol-treated fish should exhibit riskier behaviors, such as earlier emergence from the safety of a refuge, less time spent refuging and shoaling and more time spent in open habitat within an experimental arena (cf. Lima and Dill 1990; Godin 1997), compared with sham-treated controls. If cortisol-treated fish exhibit behavioral resiliency (cf. Piato et al. 2011; Schmidt et al. 2017), then there should be no differences in their behavior compared with the behavior of sham-control fish in the presence or absence of an apparent threat of predation.

## Materials and methods

### Fish collection and implantation procedures

Juvenile pumpkinseed sunfish (mean ± SE mass = 8.7 ± 0.2 g; total length = 81.8 ± 0.5 mm; *N* = 125) were captured haphazardly using a seine net in the nearshore waters of shallow weedy bays in Lake Opinicon, Ontario, Canada (44°55′90″N, 76°32′80″W) during August 2017 (Ontario Ministry of Natural Resources permit #1086180). Seining was used as the primary collection method to ensure an unbiased sample of behavioral phenotypes in the population (Wilson et al. 2011; Gutowsky et al. 2017). Following capture, fish were immediately transported to the nearby Queen’s University Biological Station (QUBS; Chaffey’s Lock, Ontario, Canada) in a well-aerated cooler and were transferred to a large, indoor flow-through tank containing lake water (∼212 L; >90% O_2_ saturation, 23.7 ± 0.1°C), where they were held for 24 h prior to experimental manipulation. A subset (*N* = 40) of the fish captured were retained for use as stimulus conspecifics for the assessment of shoaling tendency in the behavioral experiment (see below) and were not implanted with cocoa butter. These fish were held in a separate tank (∼406 L) and were kept under similar holding conditions to the focal test fish. These stimulus fish were released into the lake upon completion of the study.

**Fig. 1:**
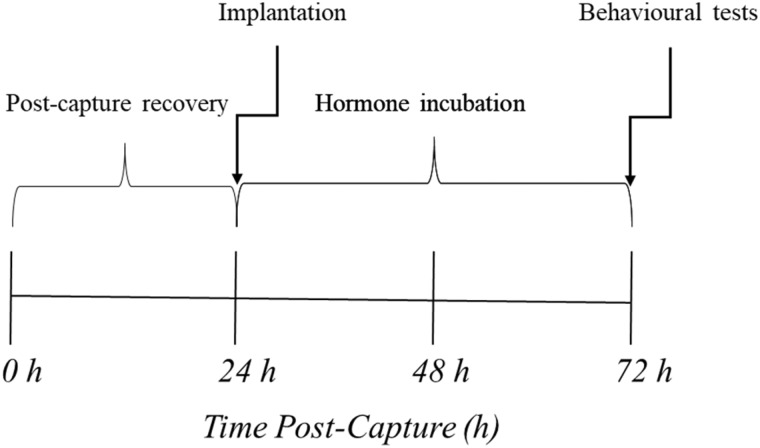
Overview of the experimental series from capture to the behavioral trials. Animals were first captured via seine net (*t* = 0 h) and were allowed to acclimate to laboratory conditions for 24 h. Following this period, individual fish were implanted with cocoa butter (*t* = 24 h) and were moved to individual holding cells where they were allowed to incubate for a total of 48 h (i.e., until 72 h post capture), as in Lawrence et al. (2018b). At 72 h post-capture, individual fish were assessed for their risk-taking behaviors in the behavioral arena. An individual focal fish was only characterized once in the behavioral arena and was lethally sampled immediately following these trials.

We captured eight Northern pike (*Esox lucius* Linnaeus; 549.6 ± 22.4 mm; range 490–650 mm), to generate predator (i.e., pike) olfactory cues used in our behavioral experiment (see below), using rod-and-reel angling techniques including trolling and bait casting (see Lawrence et al. [2018c] for more details). Upon capture, pike were transported quickly back to the QUBS and held in large outdoor tanks (∼940 L) with flow-through lake water. All pike were eventually live-released back into the lake following this study. Both pumpkinseed and pike were not fed at any time while in captivity. By the time of the onset of behavioral testing, all fish were fasted for a total of 72 h (Fig. 1). This was done to standardize individual hunger status, thereby preventing any potential confounding effects of hunger state in mediating risk-taking behaviors (e.g., Smith 1981; Dill and Fraser 1984; Gotceitas and Godin 1991). Our study conformed to the guidelines for the use and care of experimental animals of the Canadian Council on Animal Care and received prior approval of the Carleton University Animal Care Committee (AUPs #104262 and 104281).

Following a 24-h holding period, focal pumpkinseed fish were given intraperitoneal injections of cocoa butter either containing the vehicle alone as a sham control (5 mL kg^−1^ wet body weight [BW]) or suspended with cortisol (hydrocortisone 21-hemisuccinate; 25 mg kg^1^ BW). Injections were made just posteriorly to the fish’s pelvic fin using a 1 mL syringe tipped with a 16 G needle. The use of cocoa butter implants has been employed widely as a means of chronically elevating plasma cortisol titers in teleost fish (Gamperl et al. 1994), are used broadly in behavioral experiments (Sopinka et al. 2015; Crossin et al. 2016), and have been employed widely in centrarchid fishes as a means of elevating plasma cortisol titers over prolonged periods with a single dose (see Dey et al. 2010; McConnachie et al. 2012; Zolderdo et al. 2016; Algera et al. 2017a). Cortisol implants for pumpkinseed sunfish used in the current study were also validated in a separate companion study (Lawrence et al., unpublished data), which was carried out prior to the current study. Here, cortisol-treated fish exhibited significantly higher plasma cortisol titers over a 48-h sampling period than sham-treated fish (see Lawrence et al. [2018b] for further details). As in Lawrence et al. (2019), we elected to not employ a no-treatment control group here as we were interested in the relative effects of exogenous cortisol manipulation rather than the effects handling stressors associated with the implantation procedures. It is also worth noting that previous work with the closely-related bluegill sunfish (*Lepomis macrochirus*) observed no differences in plasma cortisol titers between no-treatment controls and sham-treated fish (McConnachie et al. 2012). This suggests that sham-treated fish were unlikely to be adversely affected by any stress associated with the implantation procedure. Following implantation, fish were transferred to individual blacked-out chambers (McConnachie et al. 2012; Lawrence et al. 2018b) that were maintained on a flow-through of fresh lake water. Thereafter, fish were held for an additional 48 h to ensure that plasma cortisol titers reached biologically relevant levels (McConnachie et al. 2012) prior to the behavioral trials. Following this incubation period, fish were immediately assessed for behavioral metrics associated with risk-taking behaviors (see below). A graphical representation of the time line of events from fish capture to behavioral testing can be found in Fig. 1.

### Experimental apparatus

**Fig. 2:**
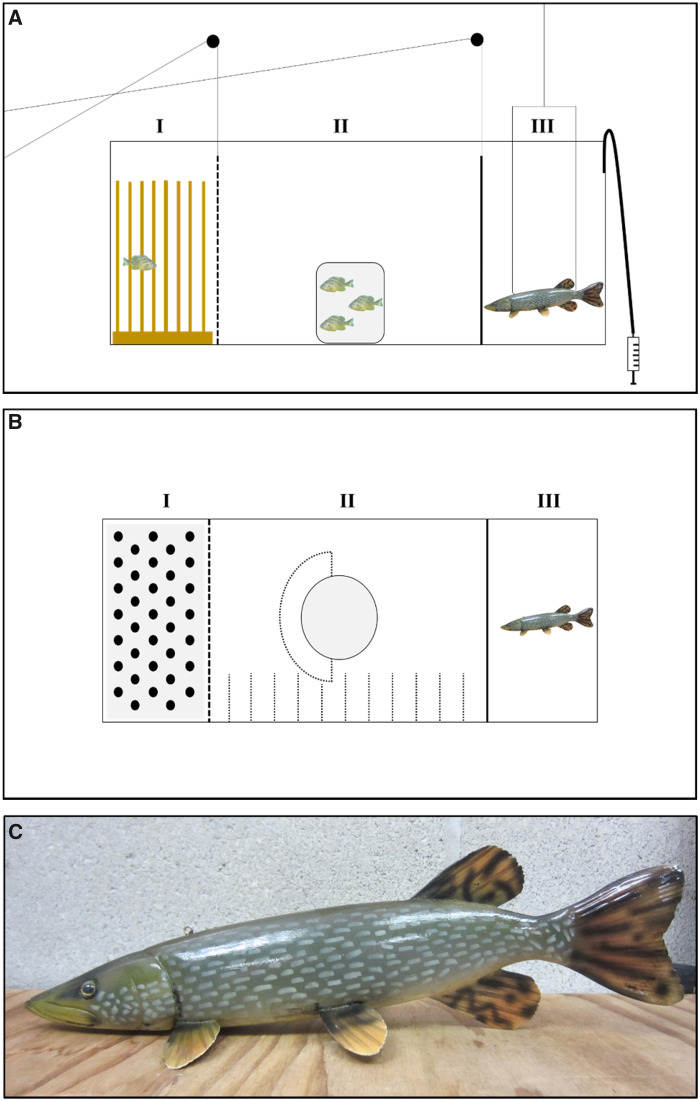
Schematic representation of the experimental arena used in the current study from a side view (**A**) and a top-down view (**B**). The experimental arena consisted of a physical refuge compartment (I) consisting of wooden dowels simulating emergent vegetation, an open zone (II) containing a stimulus shoal of three sunfish constrained in a clear glass jar, and a predator compartment (III) containing a realistic model of a pike (**C**). A clear, perforated and removable Plexiglas gate (dotted line) separated compartments I and II, and an opaque removable Plexiglas gate (solid line) separated compartments II from III. Small dotted lines in compartment II represent small stones placed on the substratum used to record the activity of focal fish in the open zone, and the dotted semi-circle around one half of the jar denotes the shoal association zone.

Behavioral trials were conducted in a standard glass aquarium (89.3 cm long × 40.6 cm wide, water depth of 28.3 cm; ∼102.6 L, Fig. 2A). The entire bottom of the aquarium was filled with white aquarium gravel (∼2 cm deep) to facilitate fish filming from overhead (see below). The experimental arena was illuminated overhead with diffuse fluorescent lighting. Additionally, all but the front side of the aquarium were blacked out to avoid potential external disturbances. The entire apparatus was enclosed within a blind, and all manipulations of the experimental arena were carried out from behind the blind. The behavioral arena was arranged in a conceptually similar manner to those used in prior works assessing risk taking in teleost fishes (e.g., Godin and Sproul 1988; Dowling and Godin 2002), and which has been previously used to assess predator fright responses in similarly-sized sunfish *L.macrochirus* (Wilson and Godin 2009) to that in our current study. The experimental arena consisted of three compartments: (i) an absolute refuge at one end (14.5 cm long × 40.6 cm wide), (ii) a central open water zone (60.1 cm long × 40.6 cm wide), which contained a constrained shoal of conspecifics, and (iii) a predator compartment at the opposite end (14.8 cm long × 40.6 cm wide), which was either left empty or contained a predator model depending on the treatment (Fig. 2A). The refuge compartment consisted of a flat piece of plywood (40 cm × 14.8 cm) that had a number of wooden dowels (1.2 cm diameter; ∼29 cm height) vertically embedded in it such that they were spaced in an offset grid pattern (3.81 cm in the X plane, 6.35 cm in Y plane; Fig. 2B). The entire structure was kept submerged through the use of adhered lead weights. This system has been used previously to simulate emergent vegetation and to provide a refuge habitat for prey fish (Mattila 1992; Dowling and Godin 2002; Snickars et al. 2004). The refuge compartment, which represented our lowest-risk habitat in this study, was separated from the rest of the tank by a clear, perforated Plexiglas (Evonik Performance Materials GmbH, Germany) partition (hereafter termed gate). The gate extended above the water’s surface and could be raised or lowered remotely from behind the blind using an overhead string and pulley system.

We placed horizontal gridlines at 10-cm intervals on the bottom (extending ∼6 cm long) and across the width of the open zone using small, gray stones (≤1.5 cm diameter) to facilitate the recording of fish activity patterns (Fig. 2A). A group of three stimulus conspecifics, constrained within a 3.78L glass jar with a perforated screw-top lid (Fig. 2A), was placed in the center of the open zone to assess the sociability of the focal fish. The side of the jar facing the predator compartment was blacked out to prevent the stimulus fish from being startled by the predator model (see below) and thereby influencing the behavior of the focal fish. On the side of the jar facing the refuge compartment, a semi-circle ring of stones (as above) was placed 10 cm from the jar. Since shoaling reduces individual risk of predation in fishes (Godin 1986; Lima and Dill 1990), we considered this semi-circle zone near the shoal as relatively safe space, but less safe than the refuge. We recorded the shoaling tendency of a focal fish as the time it spent affiliating with the stimulus shoal (i.e., within the semi-circle zone). We regarded the open zone (excluding the latter semi-circle shoal association zone) as the most risky section of the experimental arena because animals are most exposed and vulnerable to predation in open, unstructured habitats (cf. Lima and Dill 1990; Godin 1997).

The predator compartment was separated from the adjacent open zone by a clear Plexiglas partition (hereafter gate) that was blacked out and connected to an overhead string and pulley system similar to that of the refuge gate. Depending on the treatment, this compartment either was left empty (control) or contained a realistically painted model of a northern pike (310 mm TL; Fig. 2C), which was suspended in the water column ∼4 cm off of the substratum by clear monofilament fishing lines attached to an overhead anchoring point. In both treatments and just prior to the onset of the focal fish’s acclimation period, we remotely delivered an olfactory cue into the predator compartment using a 50 mL syringe and an 80-cm long piece of aquarium tubing (Fig. 2A). For the predator present treatment, the olfactory cue consisted of 50 mL of water obtained from a cooler (∼340 L) containing a single live northern pike (490–650 mm) that was allowed to sit undisturbed for 30–40 min. Presumably, the excrements and metabolites given off by the pike would generate olfactory cue(s) that would, when paired with the visual stimulus of the pike model, simulate an apparent local threat of predation to the focal fish (cf. Kats and Dill 1998; Brown 2003). This pike olfactory cue(s) was made fresh daily and stored on ice throughout the experimental day. This cue represents a concentrate of pike-derived metabolites that would not be found in such high concentrations in ambient lake water (i.e., not background levels). Thus, for the predator absent (control) treatment, the olfactory cue introduced into the predator compartment was 50 mL of lake water to serve as a control. This water was sourced from the same inflow of water entering our fish holding facility described earlier and should conceivably represent very low background levels of northern pike olfactory cues/metabolites.

### Behavioral tests

Our experiment consisted of a 2 × 2 factorial design with cortisol treatment and predation risk treatment being the two main effects. As described above, focal fish received either a cocoa butter implant laced with cortisol (25 mg kg^−1^ BW) or the vehicle alone (i.e., sham implant). The apparent predation risk treatment consisted of two levels, relatively high (predator model present paired with pike olfactory cues) or relatively low (control: predator model absent, lake water cues present). On any given experimental day, a maximum of eight focal fish were tested in the experimental arena in a balanced combination of the two main effects (i.e., two cortisol/two sham fish, two predator-present/two predator-absent control fish). Behavioral characterizations were made once per individual focal fish and always occurred 48 h post-implant, so as to standardize the cortisol/sham exposure duration. The order of the cortisol treatments was determined through systematic randomization (i.e., cortisol, sham, cortisol, sham, etc.) that was alternated on a daily basis. Individual fish were then assigned to the predation-risk treatment pseudo-randomly using a coin toss, such that there was a maximum of two focal fish in each predation-risk treatment (i.e., two predator-present trials and two predator-absent control trials). Both of these processes were in place to avoid potential treatment order biases within the study’s design.

All behavioral trials were always conducted on focal fish that were implanted for a standardized 48 h. Prior to the onset of a given experimental trial, the gates to both the refuge and predator compartments were closed. Three conspecific fish were selected haphazardly from their holding tank and transferred into the glass jar in the center of the open zone to form a stimulus shoal. These fish were used only once per day. A focal fish (48 h post-implant) was transferred from its holding tank and to the refuge compartment of the experimental arena. Great care was taken to minimize handling times and air exposure to avoid any acute stress-induced effects on fish behavior during the trial (Schreck and Tort 2016). The appropriate olfactory cue (i.e., 50 mL of pike odors or lake water) was then remotely delivered into the predator compartment. While diffusion of the olfactory cues was not measured here, we assumed that these chemical cues were contained within the predator compartment when the gate was lowered and then diffused outward into the experimental arena when the gate was raised. Focal fish were then allowed to acclimate within the refuge compartment for 30 min. During this period, the focal fish and the shoal were within sight of one another. Following the acclimation period, we started a behavioral trial by remotely raising the gate of the refuge compartment, thus providing the focal fish a choice to emerge from the refuge and enter the open zone, affiliate with the stimulus shoal, or remain in the refuge. The trial comprised a 10-min “pre-predator exposure” phase (with the predator compartment closed), followed by a 10-min “predator exposure” phase (with the predator compartment open). During the pre-exposure phase, we recorded the latency time for the focal fish to initially emerge from the physical refuge (=“refuge emergence time”), and thereafter the total times that it spent in the refuge (=“refuging time”), affiliating with the stimulus shoal (=“shoaling time”), and in the open zone (=“open-zone time”) using an overhead Go Pro Hero 3 camera (Go Pro, San Mateo, CA, USA; Struthers et al. 2015). Fish that did not emerge from the refuge for the entire 10-min phase were assigned a maximal emergence latency time of 10 min. General “activity” was scored as the number of grid lines crossed by the fish in the open zone. These behavioral measures provided a baseline level of focal fish behavior prior to its exposure to the predator compartment. At the end of this first 10-min period, we remotely raised the gate to the predator compartment, thus allowing the focal fish to view (and smell) either the predator model or an empty predator compartment. We then recorded for the 10-min predator exposure phase the behavior of the focal fish as follows.

As an immediate response to raising the gate of the predator compartment, focal fish typically either fled to the refuge compartment (*N* = 31) or associated with the stimulus shoal (*N* = 11), which we consider here a biological refuge from predation (cf. Godin 1986). Fish that initially fled to the predator compartment, fled to the shoal, or remained in the open zone when the gate was raised were not included in emergence time analysis of the post-predator exposure phase. We subsequently recorded the time taken for the fish to initially leave the physical refuge compartment and considered this latency time as the fish’s initial refuge-emergence time during this second phase. Fish that did not initially emerge from the refuge, as defined above, for the entire 10-min phase were assigned a maximal emergence latency time of 10 min. Activity score, time spent in the open zone, time spent shoaling, and time spent inside the predator compartment were only recorded for focal fish that had initially emerged from the refuge during the pre-predator exposure phase. This was done to avoid any skewing of the data set owing to those fish that remained in the refuge compartment throughout the initial 10-min pre-predator exposure phase. These latter fish (*N* = 19) were subsequently censored from the analysis. Finally, to compare the behavior of the fish between treatments, we expressed separately general activity (the number of grid lines crossed), refuging time, shoaling time, and open-zone time as difference scores (*S*_D_), calculated as the value of the behavioral measure obtained for the pre-predator exposure phase (*S*_pre_) minus its value obtained for the predator exposure phase (*S*_exp_) such that *S*_D_ = *S*_pre_−*S*_exp_.

At the end of the behavioral trial, the focal fish was removed from the experimental arena, euthanized via cerebral percussion, weighed for wet body mass (to the nearest 0.5 g), measured for total length, and its external parasites enumerated. The fish’s liver was also excised and weighed for the determination of the hepatosomatic index (HSI), following Busacker et al. (1990). Because of lethal sampling following behavioral trials, individual focal fish were only assessed once for behavioral phenotypes associated with risk-taking behaviors. The fish in the stimulus shoal were removed from the arena, placed into a separate holding tank and allowed to recover overnight. In between each successive behavioral trial, the water in the experimental arena was completely drained and refilled with fresh lake water to minimize any residual cues associated with focal fish excretions and/or the olfactory cues added to the experimental arena during the preceding trial.

### Data analyses

All statistical analyses were conducted in R Studio (Version 1.1.456; R Studio Team 2015). Statistical significance was Bonferroni corrected to *α* = 0.007 (i.e., *α* = 0.05/7) to account for the multiple behavioral measures being recorded and analyzed for individual fish (Johnson and Sih 2007). Refuge emergence times from both the pre-predator and predator exposure phases were analyzed using a Cox proportional-hazards model (package “survival”; Therneau 2015). Instances where the fish did not leave the refuge were included in the statistical model (with a maximum latency time of 10 min) but were considered as censored values in the Cox analysis. For the pre-exposure phase, the model included the main effects of implant treatment and the fish’s body mass, ectoparasite load, HSI and trial time of day as covariates. For the predator exposure phase, the model additionally included the predation risk treatment (i.e., pike model present vs. absent) as a fixed effect, the interaction between the two treatments (implant treatment × predation risk treatment), and refuge location as a covariate to account for fish who initially fled to the refuge compartment or were already in either refuge, when the predator compartment gate was raised to start the predator-exposure phase.

Difference scores for activity, total refuging time, shoaling time, and open-zone time were analyzed using separate GLMs. All models included the main effects of implant treatment and predation risk treatment and their interactive term, as well as fish body mass, ectoparasite load, HSI, trial time of day as covariates. All difference score data were fitted to a Gaussian distribution. GLMs were subjected to AIC_C_ model simplification (Hurvich and Tsai 1989; Burnham and Anderson 2002). Time spent in the predator compartment was converted to a proportion (i.e., out of 10 min total) and was analyzed using a beta regression model (package: “betareg,” V3.1-0; Cribari-Neto and Zeileis 2009, 2010).

## Results

### Refuge emergence

**Fig. 3:**
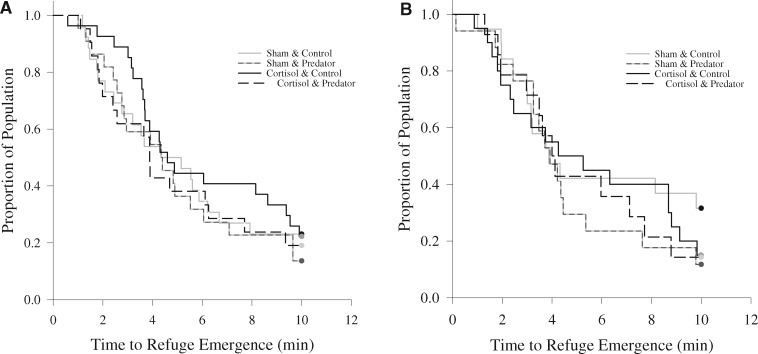
Survival curves of the latency time to emerge from refuge emergence times for focal fish during the pre-predator exposure phase (**A**) and the predator exposure phase (**B**) of the experiment for sham-control (gray lines; 5 mL kg^−1^ BW, *N* = 36–48) and cortisol-treated (black lines; 25 mg kg^−1^ BW, *N* = 34–48) pumpkinseed that were either exposed to a pike model paired with pike-derived olfactory cues (dashed lines, *N* = 31–43) or an empty predator compartment, paired with lake water olfactory cues, as a control (solid lines, left side, *N* = 39–53). Statistical significance was accepted at *α* = 0.007.

During the pre-predator exposure phase of the experiment, latency time to initially emerge from refuge was unaffected by cortisol (*z* = −0.442; *P* = 0.659) and predation risk treatments (*z* = −0.180; *P* = 0.857; Table 1 and Fig. 3A), nor was there an interaction between these two main effects (*z* = 0.434; *P* = 0.664). There were no statistical effects of any of the covariates on refuge emergence times (all *P*s > 0.007; Table 1).

**Table 1:** Summary statistics for all behavioral metrics measured

Behavioral measures		Test statistic	*P*-value
Pre-predator exposure phase			
Refuge emergence time		*z-*value	
	Cortisol treatment	−0.442	0.659
	Predation risk treatment	−0.180	0.857
	Interaction	0.434	0.664
	Body mass	−2.642	0.008
	Parasite count	0.487	0.626
	Time of day	0.749	0.454
	HSI	−1.105	0.269
Predator exposure phase			
Refuge emergence time		*z*-value	
	Cortisol treatment	0.659	0.510
	Predation risk treatment	1.224	0.221
	Interaction	−0.771	0.441
	Body mass	−0.882	0.378
	Parasite count	0.185	0.854
	Time of day	0.017	0.986
	HSI	0.560	0.575
	Refuge status	1.640	0.101
Activity		*t-*value	
	Constant	0.076	0.939
	Cortisol treatment	−0.787	0.434
	Predation risk treatment	−1.354	0.180
	Interaction	1.317	0.193
	Body mass	0.717	0.476
	Parasite count	2.065	0.043
	Time of day	−2.403	0.019
	HSI	0.006	0.996
Refuging time		*t-*value	
	Constant	−1.371	0.173
	Cortisol treatment	1.199	0.234
	Predation risk treatment	−0.423	0.674
	Interaction	−0.173	0.863
	Body mass	2.819	**0.006**
	Parasite count	1.680	0.097
	Time of day	−0.505	0.615
	HSI	0.321	0.749
Shoaling time		*t-*value	
	Constant	0.592	0.556
	Cortisol treatment	0.914	0.364
	Predation risk treatment	1.506	0.137
	Interaction	−1.582	0.118
	Body mass	−1.982	0.052
	Parasite count	−1.040	0.302
	Time of day	1.330	0.188
	HSI	−0.347	0.730
Open-zone time		*t*-value	
	Constant	1.473	0.146
	Cortisol treatment	0.497	0.621
	Predation risk treatment	−0.331	0.742
	Interaction	−0.991	0.325
	Body mass	−1.050	0.298
	Parasite count	−1.489	0.141
	Time of day	1.357	0.179
	HSI	−1.447	0.153
Time in predator compartment		*z*-value	
	Constant	−1.119	0.263
	Cortisol treatment	1.736	0.083
	Predation risk treatment	−1.403	0.161
	Interaction	−1.344	0.179
	Body mass	0.250	0.802
	Parasite count	0.563	0.573
	Time of day	1.067	0.286
	HSI	−0.364	0.716

Mains effects include cortisol treatment (cortisol vs. sham control) and predation risk (pike model present vs. absent) and the covariates included in the models (body mass, ectoparasite count, trial time of day, hepatosomatic index [HSI], refuge status). Bolded values indicate statistically significant results (*α* = 0.007). Test parameters are specific to the statistical model used, with the constant representing the *Y*-intercept of the model.

Similarly, during the predator exposure phase, latency time to emerge from refuge was not affected by cortisol treatment (*z* = 0.659; *P* = 0.510) or predation risk treatment (*z* = 1.224; *P* = 0.221; Table 1 and Fig. 3B), nor was there a significant interaction between these two main effects (*z* = −0.771; *P* = 0.441; Table 1). No other covariate (all *P*s > 0.007; Table 1) influenced refuge emergence times during the predator-exposure phase.

### Activity and spatial use patterns

**Fig. 4:**
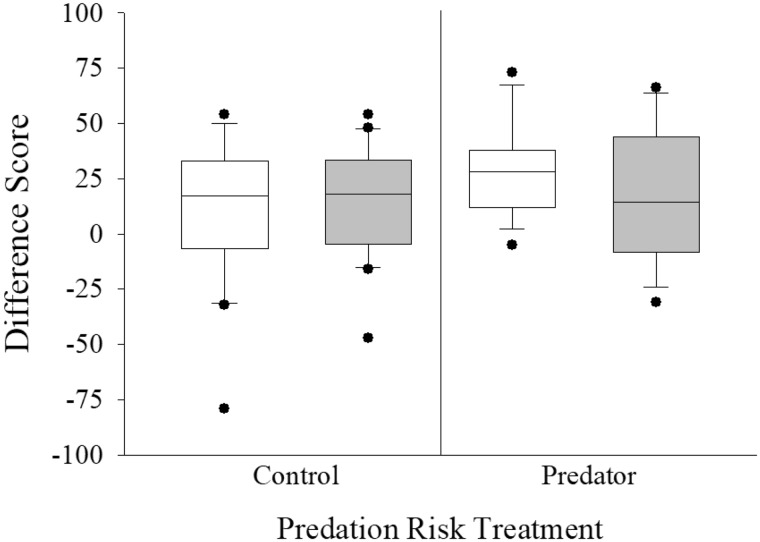
Box plot depicting difference scores for the total number of horizontal lines crossed (i.e., activity) for sham-control (white bars, 5 mL kg^−1^ BW, *N* = 37) and cortisol-treated (gray bars, 25 mg kg^−1^ BW, *N* = 37) pumpkinseed that were either exposed to a pike model paired with pike-derived olfactory cues (right side plots, 50 mL, *N* = 33) or an empty predator compartment, paired with lake water olfactory cues, as a control (left side, *N* = 41). Box plots depict the median difference score value, delineated by the interquartile range (First–third quantile) and an accompanying whisker that represents 1.5× beyond this range. Suspected statistical outliers are presented as black circles outside of the interquartile range. Statistical significance was accepted at *α* = 0.007.

Difference scores for fish activity were generally positive across all of our treatment groups, indicating that fish exhibited higher activity levels during the pre-predator exposure phase compared with the predator exposure phase (Fig. 4). However, neither cortisol treatment (*t* = −0.787; *P* = 0.434) nor predation risk treatment (*t* = −1.354; *P* = 0.180; Table 1 and Fig. 4) affected the difference scores for fish general activity. Furthermore, there was no interaction between these two main effects (*t* = 1.317; *P* = 0.193; Table 1) and none of the covariates were significant predictors of activity patterns (all *P*s > 0.007; Table 1).

**Fig. 5:**
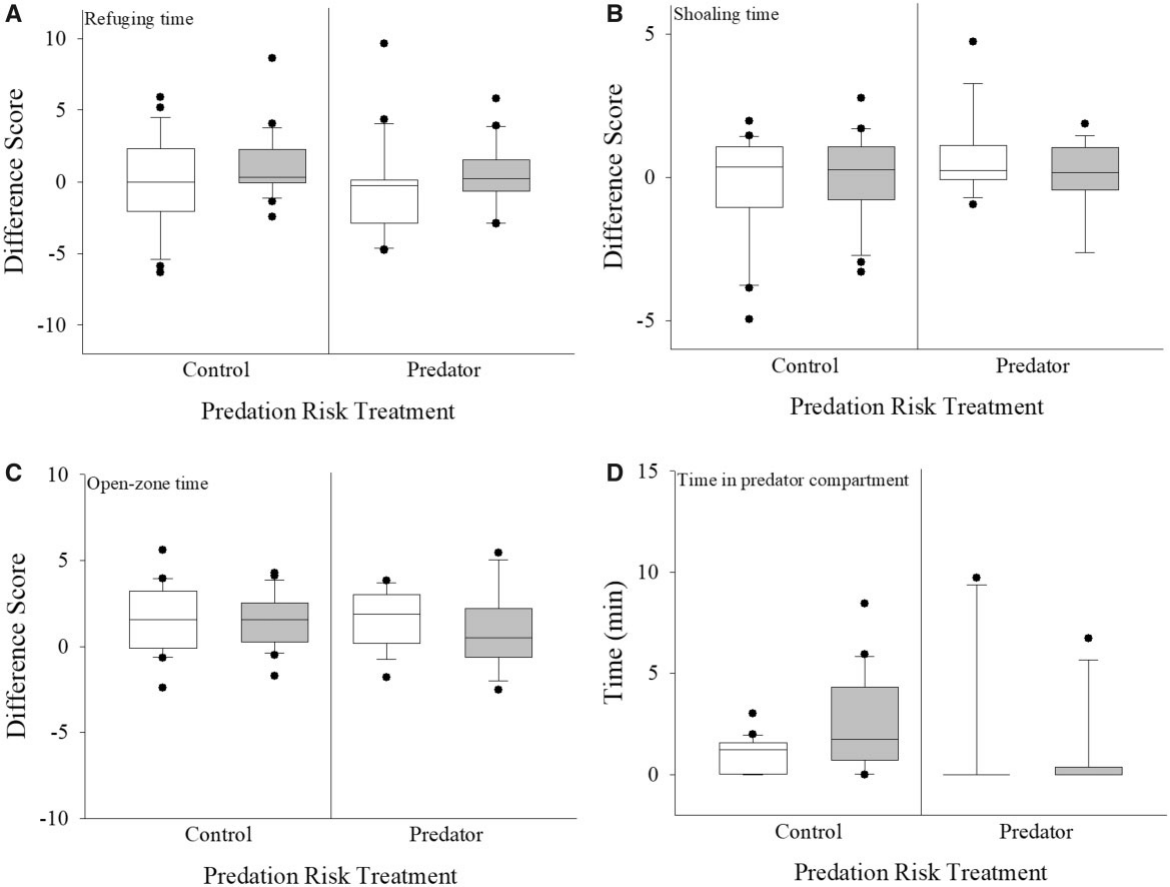
Box plots depicting the difference scores for total time spent in refuge (**A**), time associating with the shoal (**B**), time spent in the open zone (**C**), and the absolute time spent in the predator compartment (**D**) for sham-control (white bars, 5 mL kg^−1^ BW, *N* = 37–46) and cortisol-treated (gray bars, 25 mg kg^−1^ BW, *N* = 37–47) pumpkinseed that were either exposed to a pike model paired with pike-derived olfactory cues (right side plots, 50 mL, *N* = 33–40) or an empty predator compartment, paired with lake water olfactory cues, as a control (left side, *N* = 41–53). Box plots are as described in Fig. 3 caption. Statistical significance was accepted at *α* = 0.007.

Total time spent in refuge appeared to be comparable between the pre-predator exposure and predator exposure phases, as median difference scores approximated 0 (Fig. 5A). Neither cortisol treatment (*t* = 1.199; *P* = 0.234) nor predation risk treatment (*t* = −0.423; *P* = 0.674; Table 1 and Fig. 5A) affected refuging time difference scores. These two main effects did not interact statistically (*t* = −0.173; *P* = 0.863, Table 1). While most of the covariates were not statistically significant in our model (all *P*s > 0.007; Table 1), fish body mass did influence refuge use (*t* = 2.819; *P* = 0.006; Table 1).

Shoal use difference scores also generally approximated 0, suggesting that the fish exhibited comparable shoaling time during the pre-predator exposure and predator exposure phases (Fig. 5B). Shoaling was not affected by cortisol treatment (*t* = 0.914; *P* = 0.364) or predation risk treatment (*t* = 1.506; *P* = 0.137; Table 1 and Fig. 5B), nor was there an interaction between these two main effects (*t* = −1.582; *P *= 0.118, Table 1). No covariates were significant predictors of shoaling behavior (all *P*s > 0.007; Table 1).

The difference scores for time spent in the open-zone were positive for all treatment combinations, indicating that the fish generally spent somewhat less time in the supposedly risky open zone during the predator exposure phase than during the pre-predator exposure zone (Fig. 5C). However, neither cortisol treatment (*t* = 0.497; *P* = 0.621) nor predation risk treatment (*t* = −0.331; *P* = 0.742; Table 1 and Fig. 5C) influenced the time that the fish spent in the open zone of the experimental arena. The interaction of these two main effects was also non-significant (*t* = −0.991; *P* = 0.325, Table 1). No covariates significantly affected the time spent in the open environment (all *P*s > 0.007; Table 1).

Across all treatment groups, pumpkinseeds spent minimal amounts of time within the predator compartment (medians <2 min; Fig. 5D). We note that the range for this behavioral measure was relatively high during the predator exposure phase. Total time spent within the predator compartment was not affected by the cortisol treatment (*t* = 1.736; *P* = 0.083) or the predation risk treatment (*t* = −1.403; *P* = 0.161; Table 1 and Fig. 5D). All covariates, as well as the interaction of the main effects, were non-significant (Table 1).

## Discussion

### Cortisol’s influence on risk-taking behaviors

Cortisol was expected to enhance risk-taking behaviors in our experimental fish given that cortisol can increase metabolic rate (Chan and Woo 1978; De Boeck et al. 2001; O’Connor et al. 2010), and that riskier behavioral phenotypes are often exhibited by fishes experiencing higher metabolic demands and/or energetic shortfalls (e.g., Giles 1983; Godin and Smith 1988; Godin and Sproul 1988; Gotceitas and Godin 1991; Krause et al. 1998; Killen et al. 2011). In contrast to our *a priori* predictions, risk-taking behaviors in pumpkinseed sunfish were unaffected by chronic cortisol elevation and a simulated apparent threat of predation. This finding is consistent with what has been observed in other wild teleosts in this context (Cull et al. 2015; Pleizier et al. 2015; Lawrence et al. 2017, 2018a, 2018b) and initially suggests that pumpkinseed sunfish were behaviorally resilient to the physiological effects of chronic cortisol elevation (cf. Piato et al. 2011; Schmidt et al. 2017; Lawrence et al. 2018b). Behavioral resilience to systemic physiological perturbations likely represents an adaptive response maintaining behavioral phenotypes that are optimally suited to current environmental conditions, thereby maximizing individual fitness (Schreck et al. 1997; Boonstra 2013a; Noakes and Jones 2016). In our current study, risk avoidance under an apparent threat of predation likely represented the most optimal behavioral phenotype given the potential costs of activity and exposure in open habitat (i.e., predator induced mortality; Lima and Dill 1990; Godin 1997), especially given that there were no additional fitness-enhancing opportunities (i.e., foraging; see below) in the experimental arena. Consequently, being able to maintain consistent behaviors across differing physiological contexts ensures continued organismal success. Thus, we surmise that cortisol has no role in mediating predator–prey interactions in this particular context. Our results suggest that pumpkinseed sunfish have sufficient capacity to maintain behavioral phenotypes that share comparable risk burdens as sham-treated fishes. However, we remain cautious in this interpretation, as our test animals did not behaviorally respond to a threat of predation in this study (see below). Thus, we cannot conclusively demonstrate that behavior resilience, in the context of predator–prey interactions, is occurring here and re-enforces the need for further investigations of the behavioral resilience hypothesis.

While behavioral resiliency can permit an animal to cope with a physiological perturbation, the capacity to do so is limited. Therefore, it is important to highlight that, while we may have observed behavioral resiliency in our pumpkinseed, the capacity to do is likely finite (Romero et al. 2009). Consequently, it could be that the pumpkinseed had sufficient capacity to behaviorally cope over the duration of our experiment (i.e., 48 h). Indeed, under the reactive scope model, the time course effects of the “wear and tear” associated with the stress response, cortisol’s actions reduce the animal’s ability to cope over time (Romero et al. 2009). This effect is evident in chronically-stressed zebrafish (*D.rerio*) whereby behavioral coping, with respect to shoal cohesion, was observed up to 7 days into the stress protocol and becoming behaviorally compromised thereafter (Piato et al. 2011). Similar effects have been observed in cortisol-treated teleosts as well. For example, behavioral resilience was observed in creek chub (*Semotilus atromaculatus*) wherein cortisol-treatment had no effect on activity and spatial use patterns, compared with respective controls (Nagrodski et al. 2013). However, cortisol-treated chub did experience higher mortality rates than controls over a 10-day exposure period suggesting a limited capacity to cope with physiological perturbations. Similarly, parental black bass treated with cortisol implants displayed comparable nest-tending behaviors to sham-controls (O’Connor et al. 2009; Dey et al. 2010; Zolderdo et al. 2016; Algera et al. 2017b). However, cortisol treatment often resulted in higher rates of nest abandonment, suggesting that fish had a limited capacity to cope with the effects of cortisol. Together, these results indicate that perhaps the potential effects of cortisol on pumpkinseed behavior may become evident over more prolonged durations of elevated plasma cortisol levels. Thus, it would be of interest to determine if a time course for such an effect does indeed exist as well as explore some of the factors that may modulate coping capacity and thresholds in individual fish.

### Predator fright responses in pumpkinseed

The presence of a pike model failed to elicit any alterations in pumpkinseed behavior. This was unexpected as the threat of a predator generally corresponds with higher refuge use (Eklöv and Persson 1995; Gotceitas et al. 1995; Krause et al. 2000), increased shoal cohesion and social association (Rehnberg and Smith 1988; Sogard and Olla 1997; Brown and Dreier 2002; Orpwood et al. 2008), and reduced activity patterns (Lawrence and Smith 1989; Engström‐Öst and Lehtiniemi 2004; Wisenden et al. 2008; Dunlop-Hayden and Rehage 2011), all effective strategies in reducing predation risk (Lima and Dill 1990; Godin 1997). The lack of effect of an apparent risk of predation on the behavior of our pumpkinseed suggests that perhaps the pike model-olfactory cue pairing was not perceived as a significant threat of predation. This effect may be rooted in the size disparity between the focal fish and the model itself. Size-dependent perception of risk is a well-established phenomenon occurring in various sunfish species (Werner et al. 1983; Werner and Hall 1988; Shoup et al. 2003) with increasing body size corresponding to lower vulnerability to predators (Werner et al. 1983; Werner and Hall 1988; Hill et al. 2004). Based on prior works, it appears as though our focal fish size class (∼8 cm TL) was close to the threshold where sunfish exhibit a sharp decrease in predator vulnerability and a change in risk perception in the environment (Werner and Hall 1988; Hill et al. 2004). For example, large bluegill sunfish (∼10–13 cm TL) did not alter their refuge use patterns in a mesocosm setting when a live predator, a largemouth bass, was present in the experimental arena. This was not the case for small bluegill (∼6–8 cm TL) where refuging increased with the predator being present (Shoup et al. 2003). Thus, our pumpkinseed sunfish may not have perceived the pike model as a significant predation threat, which may help to explain why behavior was unaffected by predator treatment. However, there appeared to be an effect of an individual’s body mass in mediating refuge use patterns, suggesting that there is likely a perception of risk in dictating pumpkinseed behavior (cf. Dowling and Godin 2002; Brown and Braithwaite 2004; Polverino et al. 2016). We caution that these latter propositions remain speculative, as further work is needed to assess size-dependent perception of predation risk in juvenile pumpkinseed. Furthermore, it should be noted that the raising of the predator gate may have resulted in a sudden startle response in focal fish. Thus, care must be taken when interpreting the effects of the predator treatment on post-attack behavioral responses, as we cannot separate potential startle responses associated with gate opening from the predation treatment effects.

## Conclusions

We tested the behavioral resilience hypothesis which posits that an organism, in the face of significant physiological perturbations, is able to maintain a consistent behavioral phenotype in such a manner that optimizes overall fitness. To that end, we hypothesized that the metabolic effects of cortisol-treatment would result in greater risk-taking behavior in pumpkinseed sunfish and would be too great for the animal to cope with. However, refuge and spatial use patterns as well as exploratory activity were unaffected by cortisol treatment. These data suggest that cortisol has no role in mediating predator–prey dynamics in our study species. We speculate that fish are behaviorally resilient to the physiological effects of cortisol treatment over the time-frame that these observations were made (48 h post-implant) providing support for the behavioral resiliency hypothesis. Indeed, in other works, the negative effects of chronic stress become evident over more extended durations than what was used in our study (>12 days; Piato et al. 2011; Pavlidis et al. 2015). Although caution must be exercised here, as the lack of a behavioral response to the predator model-olfactory cue combination across all of our treatment groups makes it difficult to definitively conclude the occurrence of behavioral resiliency in this particular context. As behavioural coping likely aids the individual in maximizing their fitness by maintaining behaviors that are appropriate to the given context (Romero et al. 2009; Boonstra 2013a, 2013b), it would be of interest to ascertain if there exists a threshold of coping ability with pumpkinseed under cortisol treatment and a time course of such events. Our hypothesis of higher risk-taking behaviors under cortisol elevations was rooted in the relationship between the fish’s metabolism and its corresponding risk-taking behaviors which are often highly variable and contextual (Farwell and McLaughlin 2009; Biro and Stamps 2010; Killen et al. 2011, 2012, 2013; Polverino et al. 2016). Thus, it is possible that in this context, no such relationship between metabolism and behavior exists in pumpkinseed sunfish under cortisol-treatment. However, we remain cautious in some of these interpretations as pumpkinseed were not provided with foraging opportunities in our current study which has been shown to be an important feature in risk assessment studies (reviewed in Milinski 1993). Furthermore, it is possible that stressors associated with the implantation procedure may have influenced our sunfish’s behavior even in the sham-treated fish (Lawrence et al. 2018b). Thus, further work is needed to fully appreciate the role of cortisol in mediating predator–prey interactions in sunfish, particularly in the context of addressing behavioral resiliency. This would conceivably require experiments that address not only a time course of action but in also providing fitness enhancing opportunities (e.g., food) to tease apart some of the finer scale behavioral changes and decision-making processes under cortisol treatment. Furthermore, as this experiment was conducted in a microcosm setting that may limit the full expression of behavioral responses to a predation threat (Godin 1997), it would be of interest to address some of the questions in a more ecologically relevant setting to which the animal could fully engage in antipredator behaviors. Nonetheless, we suggest that, alongside prior works on the topic (Lawrence et al. 2018b, 2019), cortisol appears to have negligible bearing on predator–prey interactions in wild sunfish. There is a need to conduct similar tests on a variety of vertebrate taxa to better understand the potential generality of the behavioral resiliency hypothesis.

## Author contributions

All authors contributed to the design of the experiment. The experimental trials were conducted by M.J.L. and A.J.Z. Data analyses were performed by M.J.L., with assistance from J.-G.J.G. The manuscript was written by M.J.L., with all authors contributing to revisions.
